# Hydrogen-Rich Saline—A Novel Neuroprotective Agent in a Mouse Model of Experimental Cerebral Ischemia via the ROS-NLRP3 Inflammasome Signaling Pathway In Vivo and In Vitro

**DOI:** 10.3390/brainsci13060939

**Published:** 2023-06-10

**Authors:** Yuanyuan Du, Linyu Chen, Huimin Qiao, Lan Zhang, Lan Yang, Peipei Zhang, Jing Wang, Cong Zhang, Wei Jiang, Renhao Xu, Xiangjian Zhang

**Affiliations:** 1 Department of Neurology, Second Hospital of Hebei Medical University, Shijiazhuang 050000, China; duyuan0315@126.com (Y.D.); tujiumeicly@163.com (L.C.); qiaohuimin005@sina.com (H.Q.); zhanglan2008@126.com (L.Z.); yanglan111doc@126.com (L.Y.); zhangpeipeii@aliyun.com (P.Z.); oicongcong@sina.com (C.Z.); 2 Hebei Collaborative Innovation Center for Cardio-Cerebrovascular Disease, Shijiazhuang 050000, China; 20140127wj@sina.com (J.W.); jiangwei7270@163.com (W.J.); xrh0421@sina.com (R.X.); 3 Hebei Key Laboratory of Vascular Homeostasis, Shijiazhuang 050000, China

**Keywords:** cerebral ischemia, hydrogen-rich saline, NLRP3-inflammasome, reactive oxygen species, neuroinflammation

## Abstract

Background: Our previous research revealed that inflammation plays an important role in the pathophysiology of cerebral ischemia. The function of the NOD-like receptor protein 3 (NLRP3) inflammasome is to activate the inflammatory process. Recent findings suggest that reactive oxygen species (ROS) are essential secondary messengers that activate the NLRP3 inflammasome. Hydrogen-rich saline (HS) has attracted attention for its anti-inflammatory properties. However, the protective effect and possible mechanism of HSin brain ischemia have not been well elucidated. Methods: To test the therapeutic effect of HS, we established a mouse model of distal middle cerebral artery occlusion (dMCAO) and an in vitro model of BV2 cells induced by lipopolysaccharide (LPS). The ROS scavenger N-acetylcysteine (NAC) was used to investigate the underlying mechanisms of HS. Results: HS significantly improved neurological function, reduced infarct volume, and increased cerebral blood flow in a dMCAO mouse model. ROS, NLRP3, Caspase-1, and IL-1β expression increased after cerebral ischemia, and this was reversed by HS treatment. In BV2 cells, the application of NAC further demonstrated that HS could effectively inhibit the expression of the ROS-activated NLRP3 inflammasome. Conclusions: HS, as a novel therapeutic option, could exert protect the brain by inhibiting the activation of the ROS-NLRP3 signaling pathway after cerebral ischemia.

## 1. Introduction

Ischemic stroke refers to the sudden interruption of blood circulation in the brain and is a major cause of mortality and disability worldwide [1]. A series of pathophysiological reactions are triggered after cerebral ischemia, including excitatory amino acid toxicity, intracellular calcium overload, oxidative stress, and prominent inflammation [2]. The use of a recombinant tissue plasminogen activator as a form of traditional therapy remains problematic, yielding a poor prognosis. Therefore, we must look for new therapies to improve the treatment of ischemic stroke.

It has been proven that Hydrogen has anti-inflammatory, antioxidative, and anti-apoptotic effects [3,4]. Ohsawa et al. first reported a molecular hydrogen-induced reduction in hydroxyl free radicals and per oxy nitrite anions in a rat brain after ischemic injury [5]. Since then, hydrogen has been found to have protective effects in multiple organs [6,7,8]. Hydrogen-rich saline (HS, therapeutic dose of hydrogen dissolved in saline) is a popular mode of administration that has been shown to have neuroprotective effects against ischemic injury [8]. Recently, some studies have found that HS may affect gene expression and signal transduction pathway activation, expanding the scale of hydrogen research [3].

The inflammasome is a crucial part of the innate immune system. The NOD-like receptor family, pyrindomain-containing3 (NLRP3), is currently the most well-characterized inflammasome [9]. The activation of NLRP3 can stimulate the assembly of apoptosis-associated speck-like proteins containing the CARD domain (ASC) and pro-Caspase-1 into a cytoplasmic protein complex, known as NLRP3 inflammasome [9,10,11]. Subsequently, the activation of pro-Caspase-1 is triggered by autoproteolysis, leading to the cleavage of pro-IL-1β [11]. Recent studies have shown that increased generation of reactive oxygen species (ROS) is a key factor in the activation of the NLRP3 inflammasome [12,13]. Furthermore, some researchers have found that the ROS scavenger N-acetylcysteine (NAC) blocks the activation of the NLRP3 inflammasome [14,15]. The NLRP3 is the most extensively studied inflammasome in cerebral diseases [16,17]. Therefore, a precise understanding of the NLRP3 inflammasome is essential to find novel anti-inflammatory drugs for cerebral diseases.

However, relatively little is known regarding the potential of HS to restore cerebral injury and whether its anti-inflammatory action might participate in this effect. Therefore, the objective of this study was to confirm whether HS plays an anti-inflammatory role by inhibiting ROS, thereby restraining the NLRP3 inflammasome, and whether it can protect the brain from ischemia injury in mice.

## 2. Materials and Methods

### 2.1. Experimental Animals and dMCAO Model

Animal care and experimental steps have been described in our previous study (see [18]). This study was approved by the Animal Care and Management Committee of the Second Hospital of Hebei Medical University and conformed to the ARRIVE guidelines (Permit No: HMUSHC-130318). In general, we selected male C57BL/6 mice aged 8–12 weeks and weighing 21–25 g (Weitonghe Experimental Animal Technology Co., Ltd., Beijing, China). Animals were raised ina 12/12 h light/dark cycle, with a humidity of 60% ± 5% and a temperature of 22 ± 3 °C. All animals could freely access food and water.

Standard distal middle cerebral artery occlusion (dMCAO) was used to induce focal cortical ischemia [19]. After intraperitoneal injection of avertin (400 mg/kg, Sigma, Aldrich, Saint Louis, MO, USA) for anesthesia, a moderate incision was made at the right side of the neck to isolate the right common carotid artery and block the blood flow. Then, we used a high-speed dental drill to drill a small hole with a diameter of 2~3 mm in the skull (between the right eye and ear) to expose the distal segment of the right-middle cerebral artery (MCA). The cortical branches of the MCA were cauterized, and care was taken to avoid damaging the surrounding brain tissue. Sham-operated mice underwent the same procedure but without interruption of the blood flow or damage to the arteries. Body temperature was maintained at 37.5 ± 0.5 °C throughout the experiment.

### 2.2. Preparation of HS and Hydrogen-Rich Medium

Hydrogen-rich saline (HS) or medium was prepared as previously described [20,21]. A hydrogen generator (SPE-300, Jinan Haowei Experimental Instrument Co., Ltd., Jinan, China) was used to electrolyze water to produce H_2_ with a purity of 99.999%. H_2_ was dissolved in 0.9% saline or medium for 6 h under high pressure (0.4 MPa) to a concentration of 0.8 mmol/L. HS was freshly prepared before each time. HS concentration was determined using gas chromatography as previously reported by Ohsawa et al. [5].

### 2.3. Experimental Plan and Drug Administration In Vivo

**Figure 1:**
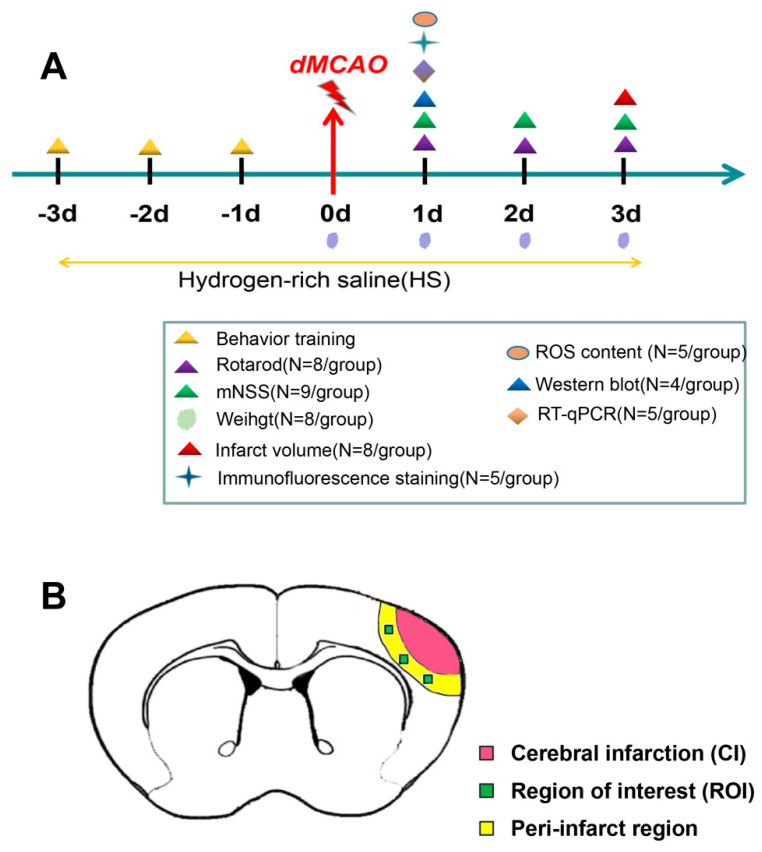
Experimental block diagram and schematic representation of brain slices. (**A**) Experimental diagram: Hydrogen-rich saline was administered intraperitoneally once daily during three days before and after the surgery. Neurobehavioral tests were performed at day 1, day 2, and day 3 after the procedure. ROS and NLRP3-inflammasome were detected at the indicated time points. The number of subjects per group for each test is shown between parentheses. (**B**) Schematic representation of brain slices. Green squares indicate the region of interest in the ipsilateral peri-infarct cortex, which is where immunofluorescence images were collected. The yellow strip (0.5 mm wide) indicates the peri-infarct region, which is where samples for RT-qPCR and western blot were obtained.

Mice were randomly divided into three groups: Sham group, dMCAO group, and Dmcao + HS group. An equal volume of 0.9% NaCl was administered in the Sham group and the dMCAO group. The dMCAO + HS group received intraperitoneal injection of HS (5 mL/kg/day or 10 mL/kg/day) for 3 days before and after dMCAO. Finally, the animals were sacrificed by quick decapitation under deep anesthesia, and the samples were taken for subsequent analyses. The experimental timeline is shown in Figure 1.

### 2.4. Neurobehavioral Test

#### 2.4.1. Rotarod Test

We used the rotarod test to evaluate the motor coordination and learning ability of mice [22]. The whole experiment was evaluated by an examiner who was blinded to the experiment allocation. Prior to surgery, the mice were trained on the treadmill for 3 days, three times a day, with a 15 min interval. We selected mice that remained on the rotarod cylinder for at least 1 min. For testing, they were placed on the treadmill and subjected to a gradual acceleration from 4 to 40 rpm for no more than 300 s. The trial ended if the animal fell off the treadmill, and the time was recorded. The rotarod test of each group was carried out three times before and after surgery at day 1 and day 3. The average time of the three tests was considered the final test outcome.

#### 2.4.2. Modified Neurological Severity Score (mNSS)

mNSS is a comprehensive test of motor, sensory, reflex, and balance, and the outcome ranges from 0 to 18 points (no deficit, 0 points; maximum deficit, 18 points).

### 2.5. Brain Infarct Volume

Cerebral infarct volume was assessed using 2,3,5-Triphenyltetrazolium chloride (TTC) staining. Six coronal sections (thickness: 1 mm) of brain tissue were stained with 2% TTC at 37 °C for 20 min and subsequently fixed with a 4% paraformaldehyde solution overnight at 4 °C. The staining was evaluated by ImageJ software and calculated by using the following equation: Percentage hemispheric lesion volume (correction of edema) = {[total infarct volume − (volume of the right hemisphere − volume of the left hemisphere)]/volume of the left hemisphere} × 100% [23].

### 2.6. Cerebral Blood Flow (CBF) Monitoring

CBF was monitored using a real-time laser speckle blood flow imager (PeriCam PSI System, Stockholm, Sweden), as we reported previously [24]. In general terms, after fully exposing the skull, the mice were fixed on a stereotaxic instrument. Then, after disinfection of the skull surface, laser speckle images were obtained through a photo detector at a distance of 10 cm from the skull. The mean perfusion level in the region of interest was measured using PimSoft 1.3 (Perimed AB, Stockholm, Sweden) before and immediately, 24 h, 72 h after dMCAO.

### 2.7. Immunofluorescence Staining

We used a freezing microtome (Thermo Scientific, Waltham, MA, USA) to obtain coronal sections (15 μm thickness) from the brain tissue with ischemic lesions. The slices were then permeated with 0.5% Triton X-100 for 20 min, blocked with 10% donkey serum at 37 °C for 1 h, and incubated overnight with primary antibodies at 4 °C. The primary antibodies were as follows: rabbit anti-NLRP3 (1:200, Bioworld Technology, Nanjing, China); rabbit anti-Caspase-1 (1:100, Affinity biosciences, Changzhou, China); and rabbit anti-IL-1β (1:100, Abcam, Waltham, MA, USA). The following day, brain slices were washed three times with phosphate-buffered saline (PBS) and incubated with secondary antibodies for 1 h at 37 °C. After three additional washes with PBS, they were treated with Fluoromount-G containing 4′,6-diamidino-2-phenylindole (DAPI; Southern Biotech, Birmingham, AL, USA) to stain the nuclei. Immunofluorescence was observed using a laser scanning confocal microscope (Zeiss LSM880, Oberkochen, Germany). ImageJ software was used to measure the number of positive cells in five different areas around the cerebral infarction [25].

### 2.8. Western Blotting

Protein isolation and subsequent step shave been previously described [26]. Briefly, protein extraction from brain tissue was performed using gene lysis buffer purchased from Apply Gene Technologies (Beijing, China), and the concentration of proteins was determined with a BCA Protein Assay Kit (Novagen, Madison, WI, USA). The classic sodium dodecyl-sulfate polyacrylamide gel electrophoresis (SDS-PAGE) method was used to analyze the protein content using the following primary antibodies: anti-NLRP3 (1:1000, Bioworld Technology, Nanjing, China), anti-Caspase-1 P20 (1:1000, Proteintech, Wuhan, China), and anti-Cleaved IL-1β (1:5000, Abcam, Waltham, MA, USA). A GAPDH antibody (1:10,000, Bioworld Technology) was used as an internal control. The relative density of the resulting bands was analyzed using an imaging densitometer (LI-COR Biosciences, Lincoln, NE, USA).

### 2.9. Reverse Transcription Quantitative Real-Time PCR (RT-qPCR)

RT-qPCR was conducted as previously described [26]. The levels of NLRP3, Caspase-1, and IL-1β mRNA were evaluated at 24 h after dMCAO. The primer sequences used in this experiment are shown in Table 1.

### 2.10. Culture and Treatment of Microglial Cells

Mouse BV2 microglial cells (1101MOUPUMC00063) were obtained from the Cell Resource Bank of China National Biomedical Laboratory. The cell culture medium used was DMEM, containing 4 mM L-glutamine, 4500 mg/L glucose (Gibco, New York, NY, USA), 10% fetal bovine serum, and 1% penicillin mixture (Solarbio, Beijing, China). The cells were cultured at 37 °C in an incubator containing 5% CO_2_. To mimic the inflammation of cerebral ischemia, the cells were stimulated with lipopolysaccharide (LPS, 1 μg/mL, Sigma, Aldrich, Saint Louis, MO, USA). To explore the mechanism of HS, NAC (Abcam, Waltham, MA, USA) was applied at a concentration of 10 nM (dissolved in DMEM) for 30 min before LPS treatment [27]. H_2_ was dissolved in DMEM at a concentration of 0.8 mmol/L. The cell cultures were divided into five groups: Sham, LPS, LPS + H, LPS + NAC, and LPS + H + NAC. The treated cells were cultured for 6 h and collected for subsequent analysis.

### 2.11. Cell Viability Assay

The viability of BV2 cells was determined using a CCK-8 detection kit (Dojindo, Kyushu, Japan) as previously described [28].

### 2.12. ROS Measurement

ROS generation was determined using a ROS assay kit (Beyotime Biotechnology, Shanghai, China) [29]. At 24 h after dMCAO, fresh cortex from the right cerebral hemisphere was collected and ground with a glass homogenizer. The homogenate supernatant was treated with DCFH-DA probe and incubated at 37 °C in dark conditions for 30 min. ROS fluorescence intensity (488 nm excitation and 530 nm emission) was measured using a microplate reader. The protein concentration of the homogenate supernatant was measured using a BCA Protein Assay Kit. ROS intensity was expressed as fluorescence intensity divided by mg of protein.

Forin vitro studies, after treatment and washes with PBS, cells were incubated with 10 μM DCFH-DA at 37 °C for 30 min in the dark. After three washes with PBS, ROS levels were detected using a microplate reader. In addition, fluorescence images of the cells were obtained using a laser-scanning confocal microscope [30,31].

### 2.13. Statistical Analyses

SPSS 21.0 software was used for statistical analysis. Data were presented as mean ± standard deviation. One-way analysis of variance (ANOVA) was used for comparisons between three groups, and the LSD test (for variables with equal variance) or Tamhance’s T2 test (for variables with unequal variance) was used for intergroup comparison. Student’s *t*-test was used to compare the TTC-positive areas between the two groups. The Kruskal–Wallis *H* test (three groups) or the Mann–Whitney *U* test (two groups) was used for non-parametric analysis. *p* < 0.05 was identified as statistically significant.

## 3. Results

### 3.1. HS Reduced Neurological Deficits

**Figure 2:**
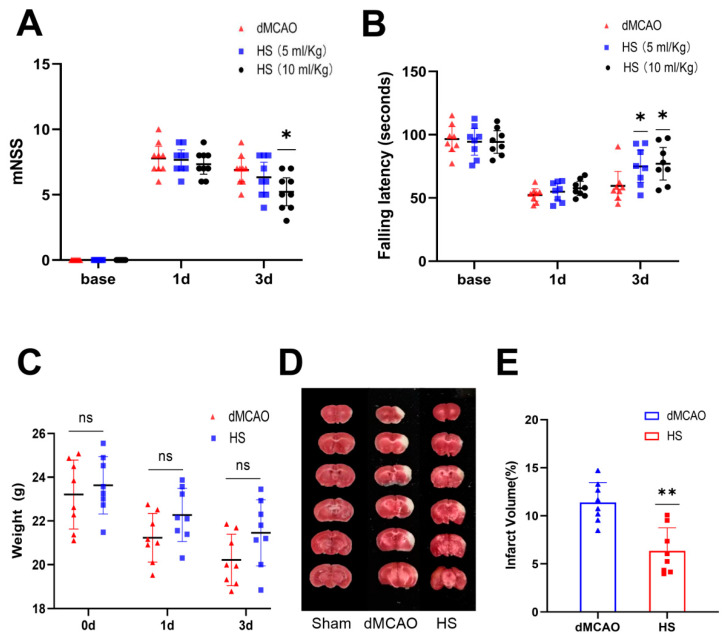
HS improved neurological recovery. mNSS evaluation (**A**), rotarod test (**B**), body weight (**C**), representative images of TTC-stained brain slices (**D**), and quantification of infarct volume (**E**). Compared with the Sham group, the neurologic function in mice from the HS-treated group (10 mL/kg) was significantly improved. They had a lower mNSS (**A**), performed better in the rotarod test (**B**), and the infarct volume was significantly reduced (**E**). N = 9 for the mNSS test, N = 8 for the rotarod test, weight, and infarct volume, * *p* < 0.05, ** *p* < 0.01, ns = no statistical difference.

We performed the rotarod test and mNSS on the mice at day 1 and day 3 after dMCAO. Higher neurological deficit scores were observed in the HS-treated and untreated dMCAO animals than in the Sham group. On day 3, there was a significant functional improvement in the HS (10 mL/kg) group compared with the dMCAO group (*p* < 0.05, Figure 2A,B), while no significant effect was observed in the HS (5 mL/kg) group in the mNSS test (Figure 2A). Therefore, treatment with HS (10 mL/kg) was selected for the experiments that followed. In general, the behavioral data suggest that HS significantly promotes neurological recovery after cerebral ischemia.

Furthermore, body weight was tested before the operation and at 24 h and 72 h after surgery. Although weight loss was less pronounced in the HS group compared to the dMCAO group, there was no significant difference between the groups (*p* > 0.05, Figure 2C).

### 3.2. HS Reduced the Infarct Volume

We measured the infarct volume using TTC at 72 h after dMCAO. Normal tissue and the infarct are a appeared dark red and pale gray, respectively. There was no infarction in the Sham group; in contrast, the dMCAO group showed extensive lesions in the cortex. The infarct volume in the HS group was significantly reduced compared with the dMCAO group (*p* < 0.01, Figure 2D,E).

### 3.3. HS Increased Cerebral Blood Flow after dMCAO

**Figure 3:**
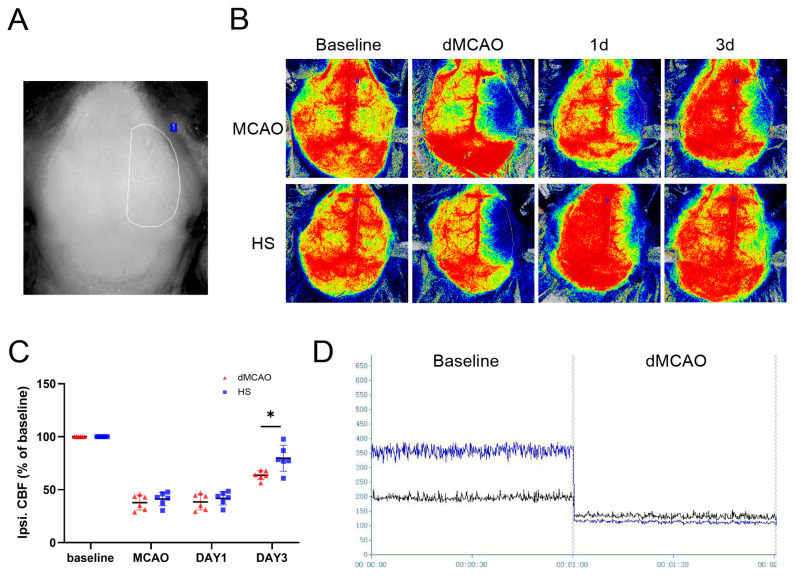
HS treatment improved cerebral blood flow after ischemic stroke. (**A**) Region of interest (Shown in white circle) in the peri-infarct region from where measurements were obtained. (**B**) Cerebral blood flow measured with laser speckle imaging in the dMCAO and HS groups before ischemic stroke and at day 1 and day 3 after ischemic stroke. (**C**) Cerebral blood flow quantification in the peri-infarct region. (**D**) Recording area in the peri-infarct region. N = 6 per group, * *p* < 0.05.

Cerebral perfusion in the right hemisphere in the dMCAO group was reduced (Figure 3A,B,D). On day 3 after operation, CBF on the lesion side had increased significantly in the HS group (*p* < 0.05, Figure 3C), whereas no improvement was observed in the dMCAO group.

### 3.4. The Cellular Location of NLRP3 and Caspase-1 after dMCAO

**Figure 4:**
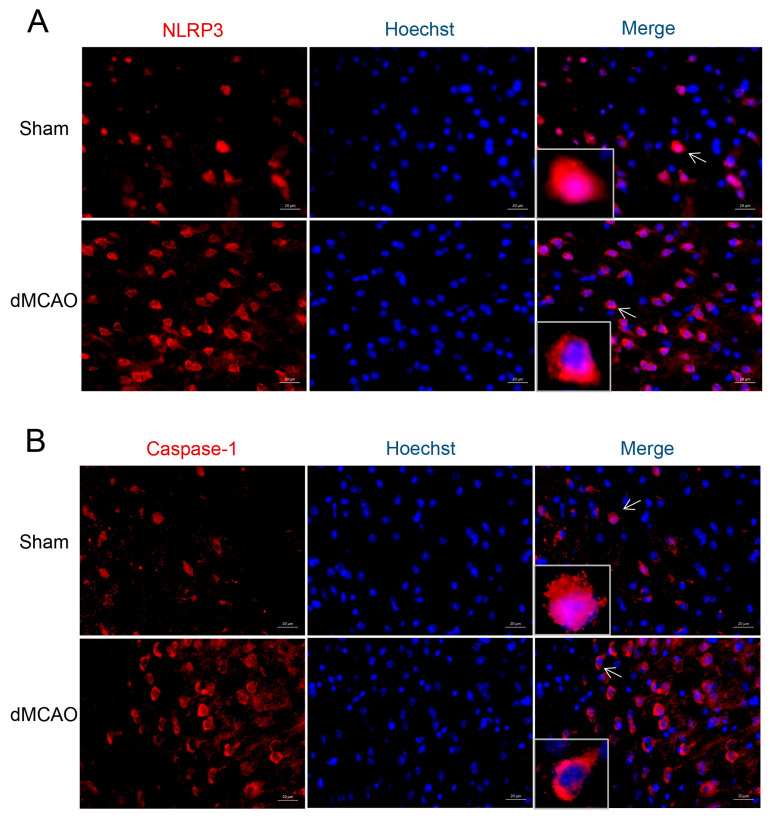
Nuclear localization of NLRP3 (**A**) and Caspase-1 (**B**) in the ischemic penumbra of the cerebral cortex. Sections were dual-stained for NLPR3 or Caspase-1 (red) and Hoechst (blue). In the normal brain (Sham), NLRP3 and Caspase-1 were mainly expressed in the nucleus, whereas they were mainly redistributed to the cytoplasm after stroke (a magnified view of the cell indicated by the arrow).

Confocal microscopy was used to detect the presence and cellular distribution of NLRP3 and Caspase-1 in the ischemic penumbra of the cerebral cortex. In the Sham group, NLRP3 and Caspase-1 were mainly localized in the nucleus. However, after dMCAO, NLRP3 and Caspase-1 were mainly redistributed to the cytoplasm (Figure 4), indicating the activation of this specific pathway.

### 3.5. HS Reduced the Expression of ROS, NLRP3, Caspase-1, and IL-1β

**Figure 5:**
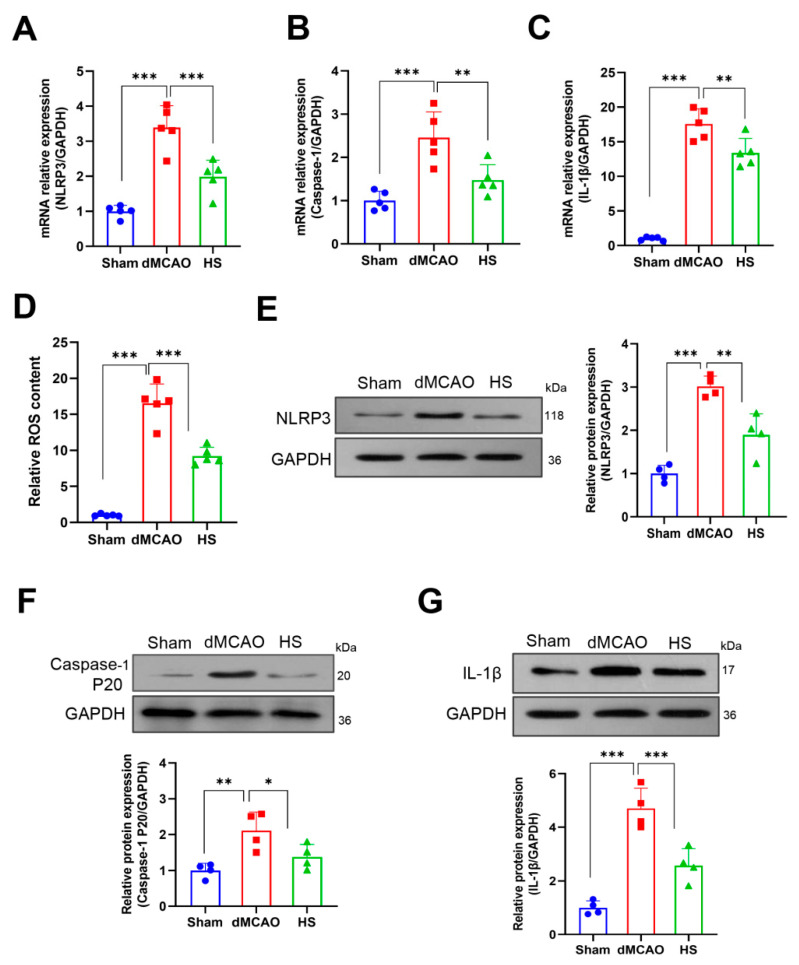
HS induced ROS production and the expression of NLRP3-inflammasome components. (**A**–**C**) mRNA expression of NLRP3, Caspase-1, and IL-1β in the peri-infarct cortex at 24 h after dMCAO. (**D**) ROS content. (**E**–**G**) Western blot and protein quantification analysis of NLRP3, Caspase-1(P20), and cleaved IL-1β in the peri-infarct cortex at 24 h after dMCAO. N = 5 for mRNA and ROS content, N = 4 for western blot, * *p*< 0.05, ** *p* < 0.01, *** *p* < 0.001.

At 24 h after surgery, ROS generation had increased. Compared to the dMCAO group, ROS levels were significantly decreased in the HS group (*p* < 0.001, Figure 5D).

**Figure 6:**
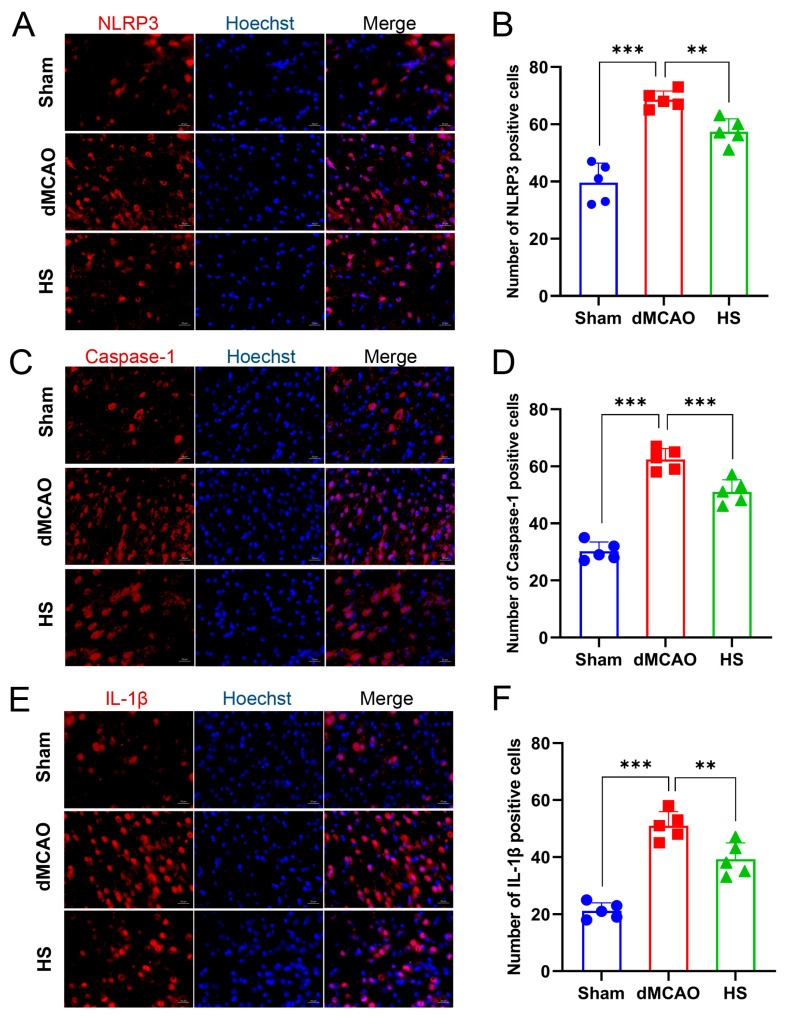
Immunofluorescence labeling of NLRP3 (**A**), Caspase-1 (**C**), and IL-1β (**E**) (red) combined with Hoechst stain (blue) in the Sham, dMCAO, and HS groups at 24 h after operation. (Scale bar, 20 μm). (**B**,**D**,**F**) quantitative analysis of NLRP3, Caspase-1, and IL-1β-positive puncta. Compared with the dMACO group, the number of NLRP3-, Caspase-1-, and IL-1β-positive puncta was significantly reduced in the HS group. N = 5 per group, ** *p* < 0.01, *** *p* < 0.001.

The expression of NLRP3, Caspase-1, and IL-1β was examined in brain tissue using RT-qPCR, western blotting, and immunofluorescence. We first determined mRNA expression at 24 h after dMCAO. The data showed that the mRNA expression of NLRP3, Caspase-1, and IL-1β was upregulated in dMCAO mice and was significantly reduced in the HS group (*p* < 0.01, Figure 5A–C). The protein expression of NLRP3, Caspase-1 (P20), and cleaved IL-1β was consistent with the results of gene expression (*p* < 0.01, *p* < 0.05, and *p* < 0.001, respectively; Figure 5E–G). The secretion of IL-1β by ELISA was markedly decreased after treatment with HS (see supplementary file). In addition, immunofluorescence analysis revealed that the positive cell numbers of NLRP3 (red, Figure 6A), Caspase-1 (red, Figure 6C) and IL-1β (red, Figure 6E) were markedly reduced in the HS group compared with the dMCAO group (NLRP3, *p* < 0.01; Caspase-1, *p* < 0.001; IL-1β, *p* < 0.01; Figure 6B,D,F). Considered together, these results suggest that HS can downregulate the production of ROS and the expression of NLRP3, Caspase-1, and IL-1βin the peri-infarction cortex after cerebral ischemia.

### 3.6. HS Exerted Anti-Inflammatory Action through Inhibiting the ROS-NLRP3 Inflammasome Pathway In Vitro

**Figure 7:**
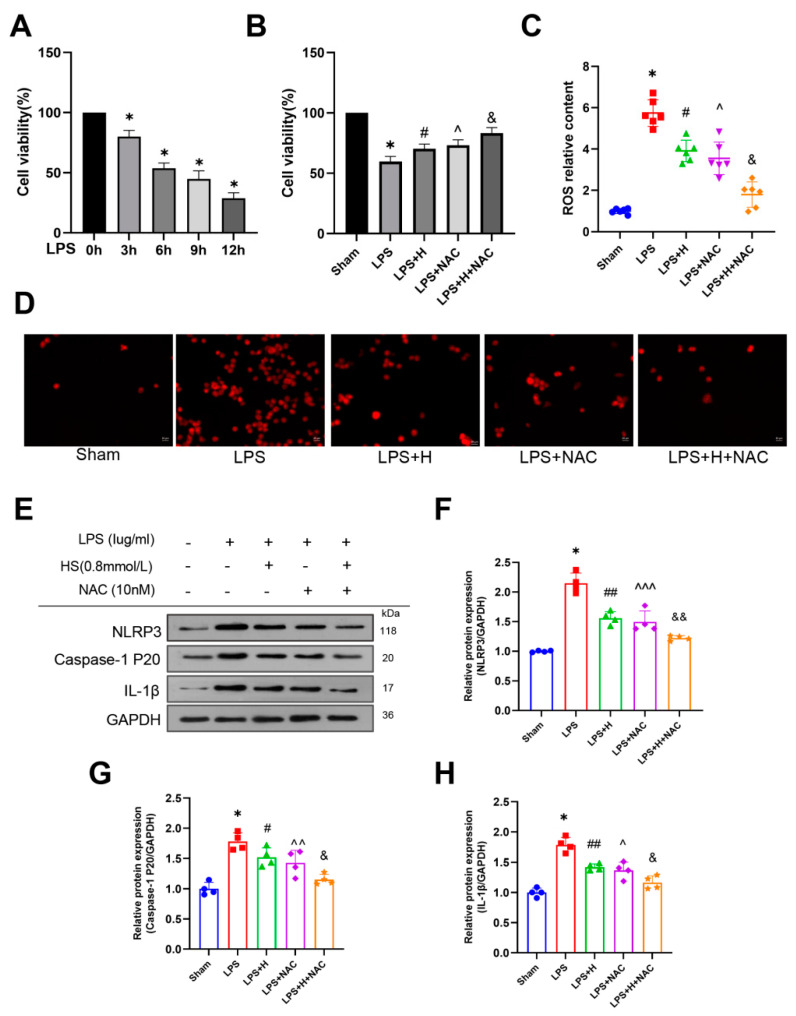
HS mediates anti-inflammatory function by inhibiting the ROS-NLRP3 inflammasome pathway. (**A**) The viability of BV2 cells after LPS treatment was detected using CCK-8 at 0 h, 3 h, 6 h, 9 h, and 12 h. N = 5 per group, * *p* < 0.05. (**B**) Cell viability of BV2 cells (N = 5 per group) and (**C**) the ROS level in the Sham, LPS, LPS + H, LPS + NAC, and LPS + H + NAC groups. * *p* < 0.001 (LPS group vs. Sham group), ^#^ *p* < 0.001 (LPS + H group vs. LPS group), ˆ *p* < 0.001 (LPS + NAC group vs. LPS group), ^&^ *p* < 0.001 (LPS + H + NAC group vs. LPS + NAC group), N = 6 per group. (**D**) DCFH-DA staining (N = 5 per group). (**E**–**H**) Protein levels of NLRP3, Caspase-1 (P20), and cleaved IL-1β in the Sham, LPS, LPS + H, LPS + NAC, and LPS + H + NAC groups. * *p* <0.001 (LPS group vs. Sham group), ^#^ *p* < 0.05 or ^##^ *p* < 0.001 (LPS + H group vs. LPS group), ˆ *p* < 0.05 or ˆˆ *p* < 0.01 or ˆˆˆ *p* < 0.001 (LPS + NAC group vs. LPS group), ^&^ *p* < 0.05 or ^&&^ *p* < 0.01 (LPS + H + NAC group vs. LPS + NAC group), N = 4 per group.

BV2 cells were treated with LPS for 0–12 h to determine the most appropriate treatment schedule. A 6 h treatment caused the death of approximately 50% of the cells in the culture and was therefore chosen for subsequent studies (Figure 7A). Our data also demonstrated that HS treatment promoted cell viability (*p* < 0.05; Figure 7B).

In order to better understand the role of HS and its potential mechanism, we detected the expression of ROS, NLRP3, Caspase-1 (P20), and cleaved IL-1β in microglial cells after LPS treatment. ROS levels rapidly increased in the LPS group compared to the Sham group, and HS reduced ROS levels (*p* < 0.001, Figure 7C). In addition, immunofluorescence staining of BV2 cells demonstrated the same trend (Figure 7D). The protein levels of NLRP3, Caspase-1 (P20), and cleaved IL-1β were upregulated in the LPS group and were down-regulated significantly after HS treatment (*p* < 0.001 for NLRP3, *p* < 0.05 for Caspase-1, *p* < 0.001 for IL-1β, Figure 7E–H). These results suggest that HS significantly reduced ROS production and the protein levels of the NLRP3 inflammasome in BV2 cells.

To further clarify this result, NAC (a ROS scavenger) was administered to BV2 cells. After NAC treatment, we observed a clear reduction in ROS levels compared with the LPS group (*p* < 0.001, Figure 7C). Simultaneously, the protein levels for NLRP3, Caspase-1 (P20), and cleaved IL-1β also decreased (*p* < 0.001 for NLRP3, *p* < 0.01 for Caspase-1, *p* < 0.05 for IL-1β, Figure 7E–H). Moreover, the combination of HS and NAC further enhanced this effect compared with the cells treated with NAC only (*p* < 0.001 for ROS; *p* < 0.01 for NLRP3; *p* < 0.05 for Caspase-1 and IL-1β, Figure 7C,F–H). The results indicate that intra cellular ROS may be involved in the activation of NLRP3 inflammasome in BV2 cells. HS effectively inhibited the expression of key components that participate in the ROS-NLRP3 inflammasome pathway.

## 4. Discussion

In the present study, we explored the neuroprotective and anti-inflammatory effects of HSon ischemia-induced brain injury in vivo and in vitro for the first time to demonstrate that (1) HS treatment contributes to the restoration of brain function by reducing infarct volume, improving neurological recovery, and increasing cerebral blood flow in a dMCAO mouse model; and that (2) HS inhibits inflammation via the ROS-NLRP3 inflammasome pathway after cerebral ischemic damage.

Ischemic stroke can lead to neurological deficits, and its incidence is increasing annually. Therefore, promoting the recovery of neurological function after ischemic stroke has become an urgent need. Hydrogen is a colorless and odorless gas that has been used to treat diseases since the 1960s [32]. Subsequently, through animal experiments, hydrogen has been found to have protective effects against injury in multiple organs, including the central nervous system, the liver, the intestines, the lungs, the stomach, the cardiovascular system, and the kidneys [33]. The application of HS is an alternative mode with the following advantages: portability, ease of administration, and safe delivery of H_2_ [6]. These pieces of evidence suggest that HS may be an attractive candidate for clinical use. Therefore, HS was chosen as the therapeutic measure in the present study.

Cerebral ischemia can trigger multiple pathological processes, such as ROS production, oxidative stress, neuroinflammation, mitochondrial injury, and neuronal apoptosis, ultimately aggravating nerve damage [34,35,36]. Considering the mechanisms of ischemic brain injury, the inhibition of excessive ROS production and neuroinflammation in the early stages is a promising treatment strategy to improve patient prognosis [37]. Anti-inflammation, accompanied by anti-oxidation, is an important mechanism of hydrogen therapy [3]. HS has been reported to have a protective role against neurodegenerative and cerebral vascular diseases [38,39]. However, the mechanism of HS is still unclear. Takeuchi et al. [40] found that hydrogen water could protect the blood–brain barrier and brain function by reducing the generation of ROS and preventing the activation of matrix metalloproteinase-9 in the hippocampus. In the piglet model of hypoxic-ischemic encephalopathy, the application of H_2_ reduced oxidative stress and promoted nerve function [41]. HS treatment significantly decreased the levels of NF-κB, TNF-α, IL-6, MDA, and Caspase-3 and markedly demonstrated protection with respect to cerebral ischemia-reperfusion in rats [8]. Chen et al. demonstrated that HS treatment attenuates the inflammatory reaction and inhibits microvascular endothelial cell apoptosis through the PI3K/Akt/GSK3β signaling pathway in a cardiopulmonary bypass-induced brain injury model [42]. In our study, we have shown that the systemic administration of HS was effective for treating neurological impairment, as it decreased infarct volume and improved cerebral perfusion in a mouse model of dMCAO. These results indicatethat HS has a definite and extensive protective effect against brain injury.

One of the most important features in the host’s defense system is the NLRP3 inflammasome [43,44]. Mounting evidence has shown that the NLRP3 inflammasome can cause inflammatory reactions linked to several diseases of the nervous system, including cerebral hemorrhage [45], brain infarction [24], traumatic brain injury [46], Alzheimer’s disease [47], and encephalitis [48]. Studies have shown that the activation of the NLRP3 inflammasome may be related to ROS production [15,49]. In the present study, we investigated the role of the ROS-NLRP3 inflammasome in ischemia-induced inflammation in vivo and in vitro. We showed that the expression of ROS, NLRP3, Caspase-1, and IL-1β increased simultaneously and that it was inhibited by HS treatment. For the in vitro test, we selected the BV2 cell line because it is widely used to study inflammatory responses [10,50]. We established our inflammatory model using LPS treatment, as described in our previous study [25]. In agreement with animal studies, HS treatment reversed the LPS-induced increase in ROS and NLRP3-inflammasome activation. Moreover, the translocation of NLRP3 and Caspase-1 from the nucleus to the cytoplasm proved the activation of the NLRP3 inflammatory pathway, while HS treatment could partially inhibit the activation of this pathway. In summary, our results revealed that cerebral ischemia induced the upregulation of ROS and NLRP3, Caspase-1, and IL-1β expression, whereas HS reduced the levels of all these factors.

ROS production is crucial for the regulation of innate immune responses. The activation of the NLRP3 inflammasome by excessive ROS production can trigger the conversion of pro-IL-1β to activated IL-1β, which drives inflammatory responses [50]. Li et al. demonstrated that intracellular ROS levels were elevated in Helicobacter pylori-infected THP-1 cells, and that this could be prevented by NAC treatment, thus inhibiting NLRP3 inflammasome activation [51]. In a mouse model for dry eye, increases in ROS production triggered NLRP3 inflammasome formation and activation, leading to the increased secretion of bioactive IL-1β [52]. Meanwhile, studies have found that excessive ROS generation can also activate the NF-κB inflammatory pathway, leading to reciprocal effects on the NLRP3 inflammasome [53,54]. Shao et al. found that elevated ROS and the activation of the NF-κB pathway and NLRP3 inflammasome after subarachnoid hemorrhage (SAH) can lead to inflammation and nerve injury by regulating the expression of pro-inflammatory cytokines and maturity of IL-1β. However, HS treatment could attenuate the inflammatory response by inhibiting NF-κB activation and NLRP3 inflammasome formation in early brain injury (EBI) after SAH [54]. This study demonstrated the beneficial effect of HS treatment similar to ours. However, in our in vitro study, the ROS scavenger NAC was used to further elucidate the mechanisms of HS. Our data showed that NAC significantly inhibited the upregulation of NLRP3, Caspase-1, and IL-1β, implying the involvement of ROS in triggering the activation of the NLRP3 inflammasome. Furthermore, co-treatment with HS and NAC further enhanced the effect observed in response to NAC alone. Overall, our results suggest that HS suppresses the activation of the ROS-NLRP3 inflammasome signaling pathway and exerts neuroprotective effects after ischemic stroke. We did not investigate the NF-κB pathway in this experiment, and the interaction between the NF-κB and the NLRP3 inflammasome is currently unclear, requiring further study.

At present, animal experiments have confirmed that the use of hydrogen can provide potential treatments for a variety of diseases [33]. Researchers have begun to conduct a number of clinical studies, showing that hydrogen has beneficial effects on cardiovascular diseases, metabolic diseases, neurodegenerative diseases, and tumor adjuvant therapy [55]. People are also concerned about the acute/sub-acute toxicity of hydrogen molecules. Hydrogen can be absorbed through inhalation, oral administration, and as a topical application. The results of these studies confirmed the safety of introduction hydrogen into the human body, showing no significant toxic effects [55,56,57]. Therefore, hydrogen has high value in terms of clinical application. However, more systematic clinical trials should be conducted to deepen the understanding of this enticing gas. New research developments are highly anticipated.

In conclusion, our study demonstrated that HS can promote neurological functional recovery through the ROS-NLRP3 inflammasome signaling pathway after dMCAO in mice. Due to the unique advantages of hydrogen, HS can hopefully provide a novel therapeutic option for the early stages of ischemic disease. Further evaluations are needed for clinical tests to clarify the potential of HS as an effective medicament for the prevention and treatment of stroke.

## Figures and Tables

**Table 1:** PCR primers used in this study.

Gene	Primers	Sequences
NLRP3	Forward	5′-CCAGCCAGAGTGGAATGACA-3′
	Reverse	5′-AGCGGGAGACAAATGGAGAT-3′
Caspase-1	Forward	5′-GGGACCCTCAAGTTTTGCC-3′
	Reverse	5′-GACGTGTACGAGTGGTTGTATT-3′
IL-1β	Forward	5′-AACTCAACTGTGAAATGCCACC-3′
	Reverse	5′-CATCAGGACAGCCCAGGTC-3′
GAPDH	Forward	5′-AGGAGCGAGACCCCACTAACA-3′
	Reverse	5′-AGGGGGGCTAAGCAGTTGGT-3′

## Data Availability

The datasets generated during and/or analyzed during the current study are available from the corresponding author on reasonable request.

## Supplementary Materials

The following supporting information can be downloaded at: https://www.mdpi.com/article/10.3390/brainsci13060939/s1, Figure S1: HS inhibits the secretion of IL-1β in vivo by ELISA at 24 h after dMCAO. *** *p* < 0.001. N = 6 per group.
