# Supplementary material for: Hydrogen-Rich Saline—A Novel Neuroprotective Agent in a Mouse Model of Experimental Cerebral Ischemia via the ROS-NLRP3 Inflammasome Signaling Pathway In Vivo and In Vitro
Source: Brain Sci. 2023 Jun 10;13(6):939. doi: 10.3390/brainsci13060939 (PMC10296247; doi:10.3390/brainsci13060939)
Supplement: Supplementary file 1 [file brainsci-13-00939-s001.zip › brainsci-2422998-supplementary.pdf]

Enzyme-Linked Immunosorbent Assay (ELISA)

The cerebral cortex on the ischemic side was isolated at 24 h after operation. The samples were added with ice-cold saline at 4 °C at a weight of 1:9 and then homogenized. Tissue homogenates were centrifuged at 4000 r/min for 15 minutes and the supernatant was obtained. The concentration of IL-1β were detected using Mouse IL-1β Valukine ELISA Kit (EK0394,Boster, Wuhan, China) following the instructions of the kit.

Result:

The secretion of IL-1β was significantly increased in the dMCAO group compared to the Sham group, and was markedly decreased after treatment with HS ($p < 0.001$).

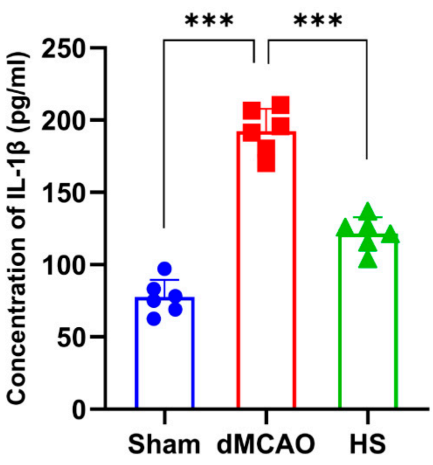

Figure legend:

Figure S1: HS inhibits the secretion of IL-1β in vivo by ELISA at 24 h after dMCAO. *** $p < 0.001$. N = 6 per group.
